# Erratum

**DOI:** 10.4269/ajtmh.66-706err

**Published:** 2022-10-10

In “Progressive Chagas cardiomyopathy is associated with low selenium levels” by Rivera and others (doi.org/10.4269/ajtmh.2002.66.706) one of the author’s names is misspelled. The third author, Alejandro M. Hasslocher Moreno, should have been listed as Alejandro Marcel Hasslocher-Moreno.

